# Supplementary material for: Leaf arrangements are invalid in the taxonomy of orchid species
Source: PeerJ. 2017 Jul 21;5:e3609. doi: 10.7717/peerj.3609 (PMC5522722; doi:10.7717/peerj.3609)
Supplement: Appendix S1 [file peerj-05-3609-s001.docx]

**Appendix A. List of examined herbarium specimens of *Epipactis purpurata*, only with leaf aberrations.**

--- **Denmark** (C Herbarium)**.** 1882 *B. Hjorth* *s.n.* (C 2/2015|10); Distr. 13a: Haslund, 18 August 1962 *H. Bach s.n.* (DK–0005410); Haslund skov, 12 August 1973 *H. Skovgaard Christensen* *s.n.* (DK–0005402); Haslund skov 13 August 1973 *S. Grove s.n.* (DK–0005389); Distr. 24: Fakkegrav, 09 August 1969 *H. Knutz s.n.* (DK–0005409); "Rosenvold"09 September 1952 *A. Hansen s.n.* (DK–0005384); Svendsmølle ved Daugaard, 17 August 1950 *S. Andersen* *s.n.* (DK–0005381). --- **England.** Surrey: Chelsham, 16 August 1953 *D.P. Young s.n.* (C 2/2015|9). --- **Germany.** Baden-Württemberg: Hohenzollern, Buchenwälder bei Sigmaringen, 20 August 1915 *Dr Hanz s.n.* (B 10 0591188, M–0257871); Schwarzwald, Wutachtal, Räuberschlößle, 24 August 1953 *D.E. Meyer 306* (B 10 0591222); Bayern: Allgäu, Wald links der Iller südöstlich Reicholzried, 03 September 1997 *E. Dörr s.n.* (M–0257860); In schattigen Bergwäldern auf Lehmboden um Waging (b. Gessenberg), Laufen (Hauniberg), Prien (Ratzinger-Berg), August 1866 *Progel s.n.* (M–0257857); München, zwischen Garatshausen und Feldafing, *H. Dihm* *s.n.*(M–0257845); Neuburg a.d. Donau, 24 August 1904 *W. Gugles s.n.* (M–0257864); Rhön, Hillenberg, 18 August 1907 *Brade s.n.* (FR–0118354); Wessling, Altinger Buchet, Fichtenwald, 23 July 1950 *H. Merksmüller s.n.* (M–0257853); westlich Schloß Eisenburg, 05 August 1996 *E. Dörr s.n.* (M–0257837); Hessen: Edelsberg, Weg nähe Grillhütte, 07 July 2004 *K. Baumann 4/419* (FR–0118340); Ober. Aula Ks. Ziegenhain, 26 July 1964 *A. Nieschalk, W. Korbach 931* (FR–0001005); Oberelsungen Ks. Wolfhagen, Indenberg, 01 September 1968 *A. Nieschalk, W. Korbach 929* (FR–0001004); Thüringen: Harz, Kohnstein, 17 August 1895 *Vocke s.n.* (Z–000088610); Hochberg, 1980 *E. Koch s.n.* (B 10 0591214); Jena, Isserstedt, 08 August 1907 *M. Schulze s.n.* (M–0257881); Legefeld (Itengstbachtal) obere Waldrand gegen d. Herlitzenberg, Laubwälder, September 1918 *J. Bornmüller s.n.* (B 10 0591230); Weimar, Ettersberg, 20 August 1916 *J. Bornmüller 9* (S14–50598); Weimar, Ettersberg, 30 August 1894 *J. Bornmüller s.n.* (B 10 0591203). **--- Poland.** Dolny Śląsk: Lwówek Śląski, Proszowa [Schlesien: Löwenberg, Gebüsch des Kalkerberges bei Kunzendorf], August 1873 *E.F. Dresler s.n.* (M–0257888); Rudna, Nieszczyce, 01 August 2016 *E. Żołubak, A. Jakubska-Busse s.n.* (WR SN 059953). **--- Switzerland**. Aargau: Rainwald nördlich Habsburg, nördl. Pt. 478, alt. 470 m, 01 August 1959 *H.U. Stauffer s.n.* (Z–000088596); Schiltwald, 24 August 1926 *P. Müller 399* (Z–000088587); Basel: Hinter Helfenberg bei Langenbruck, August 1902 *G. Müller s.n.* (ZT–00071794); Schaffhausen: Wilchingen, Wald südlich Taubental, 04 July 1995 *G. Kummer s.n.* (ZT–00071817); Seeland: Dreihubelwaldin der Hardern bei Lyss, alt. 510 m, 02 August 1924 *W. Ldi-Bern s.n.* (ZT–00071797); Solothurn: Rüttenen, Vorberg, am Weg nach Kuchigraben, 05 September 1936 *M. Brosi s.n.* (ZT–00071819); Zürich: am Käferberg-E.-Hang ob. "Guggach", alt. 515 m, 28 July 1951 *W. Koch 51/546* (ZT–00071775); Horgen, Sihlwald, Roregg, Grat, der von Pt. 680 nach Nordosten geht, 07 October 2000 *E. Landolt J24* (ZT–00071751); Langnau, südöstlich der Hochwacht, alt. 870 m, 03 October 2001 *E. Landolt G20* (ZT–00071754); Nörd. Haus ob. Zumikon, Nadelwald, alt. 720 m, 03 September 1921 *A. Thellung s.n.* (Z–000088529); Seetals 16 August 1908 *J. Meier s.n.* (Z–000088592); Stallikon, auf dem Grat, der zw. Weidel und Hagni ins Tal führt, alt. ca. 740 m, 31 July 1994 *E. Landolt s.n.* (ZT–00071744); Stallikon, westl. Station Uetliberg, alt. 760 m, 18 September 1991 *E. Landolt s.n.* (ZT–00071736); Waldrand östl. Felsengrd. Wetzwil, Pfannenstiel, 23 August 1970 *W s.n.* (Z–000088532); Zürichberg, 15 August 1933 *G. Défago s.n.* (ZT–00071810); Zürichberg, August-September 1927 *J. Bär s.n.* (S14–50602); Zürichberg, Mischwald im "Oberholz" hinten dem Alkoholfreien Kurhaus, 28 August 1926 *A. Thellung s.n.* (Z–000088572).
